# Association Between Belief in Medicine and Adherence to Antiretroviral Therapy Among Human Immunodeficiency Virus Adults in Zaira, Kaduna State, Nigeria

**DOI:** 10.7759/cureus.36489

**Published:** 2023-03-21

**Authors:** Rosemary A Eze, Norhasmah Sulaiman, Zulfitri 'Azuan Mat Daud, Aliyu Babadoko

**Affiliations:** 1 Department of Nutrition, Faculty of Medicine and Health Sciences, Universiti Putra Malaysia, Serdang, MYS; 2 Department of Dietetics, Faculty of Medicine and Health Sciences, Universiti Putra Malaysia, Serdang, MYS; 3 Department of Haematology, Ahmadu Bello University Teaching Hospital, Zaria, NGA

**Keywords:** zaria, nigeria, kaduna state, belief for medicine, human immunodeficiency virus, antiretroviral therapy

## Abstract

Background: Negative perceptions of antiretroviral treatment (ART)'s efficacy and consequences may operate as roadblocks to adherence. This research aimed to determine the association between belief in medicine and adherence to antiretroviral therapy among HIV adults on ART in Ahmadu Bello University Teaching Hospital, Zaria, Kaduna State, Nigeria.

Method: Using a cross-sectional design, a systematic random sampling method was used to select respondents aged 18-64 years on antiretroviral therapy for at least six months at Ahmadu Bello University Teaching Hospital, outpatients of the President's Emergency Plan for AIDS Relief Clinic. Socio-demographics, belief in medicine and adherence to ART were obtained using a self-administered questionnaire. Descriptive statistics, chi-square test, and multiple logistic regression were used for data analysis.

Results: Among the 385 people who took part in the study, about 67.5% were females and 32.5% were males. About 54% of adults adhered to ART. More than half (55.8%) of the respondents had negative perceptions (beliefs) of personal need for ART medication and about 42.3% of the respondents had more concerns about the potential negative effects of the ART medication. Government employment (odds ratio (OR) = 2.842, *p* = <0.01), self-employment (OR = 2.6, *p* = <0.001), and being divorced or widowed (OR = 2.0, *p* = <0.01), negative perceptions (beliefs) of personal need for the ART medication (adjusted OR (AOR) = 1.525, *p*=<0.01) and more concerns about the potential negative effects of the ART medication (AOR= 1.362, *p* = <0.05) were all significantly associated with ART adherence.

Conclusion: Employment, marital status and belief in medicine were associated with adherence to ART. Therefore, during adherence counseling, healthcare personnel should address respondents' false views and fears regarding ART medication in order to strengthen proper information and the benefits of ART.

This article was previously presented as a meeting poster at the 36th Scientific Conference of the Nutrition Society of Malaysia (7th and 8th September 2021).

## Introduction

Nigeria is ranked second in sub-Saharan Africa, behind South Africa, and third globally, behind India, in terms of illness burden, according to the United Nations [1]. In 2019, 1.9 million Nigerians were living with HIV/AIDS, with a nationwide prevalence of 2.8% and a prevalence of 1.4% among adults. However, within the same period, there was an increase in new HIV infections from 120,000 to 130,000, 53% in treatment and 42% in the number of people living with HIV viral suppression [2]. However, within the year 2020, Nigeria had 1.7 million HIV-positive persons and 49,000 persons died as a result of AIDS in that year, including both adults and children. With 20,000 deaths, male adults were the group with the highest number of deaths [3]. This might be due to the global pandemic that affected the world and as a result insufficient antiretroviral treatment (ART) medication supplies in the country.

Adherence is defined as a patient's ability to stick to a treatment plan, take drugs at the prescribed times and frequencies, and adhere to dietary and other prescription limitations [4]. Adherence refers to the capacity to take prescribed drugs according to the indicated dosages and timings, as well as any additional instructions, such as taking them on an empty stomach or after meals [5]. Since its inception in 1996, ART has considerably reduced HIV/AIDS-related mortality and morbidity [6,7]. In improving HIV/AIDS patients' prognosis and quality of life, and reducing diseases progression and death, ART has been an important part [8]. More than 90% compliance is required for older treatments, but this number is lower (80%) for newer ART regimens [9].

People's perceptions of their own motivation, mental processes, emotional states, and behavioral patterns are all part of perception. A mixed-method study done in Nepal shows the qualitative result of one participant who stated: People in rural areas continue to doubt that antiretroviral therapy (ART) works for HIV patients. People with HIV will die sooner or later, whether they use antiretroviral therapy or not. Why should I perish because I took these poisonous pills? After starting treatment, they stopped taking the drug [10]. Treatment might be aided or hampered by a patient's beliefs about HIV. Non-adherence has been linked to a lack of understanding about HIV and the consequences of ART [11]. Concerns about side effects and the assumption that once you are well, you can stop taking ART are two previously found ART beliefs in sub-Saharan Africa [12,13]. Patients may employ traditional medicines as a result of their concerns. Beliefs about ART should be identified since they affect how and/or how much medication is used and patients' opinions of the benefits of taking medication have a big impact on how much they take it [14]. Medicine beliefs, whether positive or negative, have an impact on medication usage. Patients' reservations regarding their own personal need for ART can be alleviated by providing the medical justification for ART in a way that addresses or coincides with a patient's common sense perspective. Side effect concerns can be addressed during consultations by routinely discussing any unpleasant affects they may be experiencing, as well as offering necessary pharmaceutical therapy and advice that is individualized and suited to the individual.

There are two main themes in patient beliefs about medication, according to the researchers: 1) the necessity of prescribed medication for present and future health, and 2) concerns about side effects [15]. To explain how these beliefs influence adherence, Horne et al. suggested a cost-benefit paradigm, in which necessity beliefs are evaluated against concerns [16]. Adherence is more likely if a person's belief in the necessity of their actions outweighs their fears. This framework has been tested in a range of diseases, including HIV [17,18]. In addition, people may take traditional medications because of their fears [19]. It is important to identify people's beliefs regarding ART since they can influence how the drug is taken and how much is taken [20]. To avoid adverse effects, for example, these beliefs may lead to intentional or inadvertent non-adherence with the recommended treatment plan (e.g. forgetting). Non-adherence may be the result of a patient's "informed choice," in which health care providers should engage with and understand their patients' choices in order to encourage effective decision-making among themselves. Studies done in Nigeria on sociocultural factors impacting adherence to ART are examples of studies that have looked at ART barriers and facilitators in Nigeria [21-23].

No earlier research has examined the unique beliefs of people with HIV about ART in Zaria. Analysis of the elements that influence patients' belief in treatment is critical to developing effective adherence strategies in this environment. The purpose of the study was to examine the relationship between adherence to ART and belief.

## Materials and methods

Study design and setting

The study was conducted at Ahmadu Bello University Teaching Hospital Zaria (ABUTH), Kaduna State, Nigeria. Kaduna State is one of the 36 states in Nigeria, with the capital being Kaduna. In Nigeria, the top five states with highest HIV prevalence are Akwa lbom (5.5%), Benue (5.3%), Rivers (3.8%), Taraba (2.9%), Anambra (2.4%) and Kaduna (1.1%) [24]. An 18-month programme to increase the number of people receiving ART in nine Nigerian states was launched in April 2019 by the U.S. President's Emergency Plan for AIDS Relief (PEPFAR). Despite the COVID-19 pandemic, the programme resulted in 208,202 additional HIV-infected people receiving ART (including 97,387 additional people from March 31, 2019 to March 31, 2020 and an additional 110,815 additional people from April 2020 to September 2020) [25]. However, the present study location in Kaduna state was not one of the nine states that was selected for the study. Using purposive sampling ABUTH was selected in order to determinants and prevalence of adherence to ART. The study was done at the HIV/PEPFAR clinic at ABUTH, Zaria, one of the 11 hospitals in Kaduna State, Nigeria, that gives antiretroviral therapy to HIV patients. The study was conducted from June until September 2019.

A cross-sectional study with a quantitative approach was conducted at the PEPFAR clinic of ABUTH, Zaria, Kaduna State, Nigeria. All adults aged 18 to 64 years living with HIV on ART at ABUTH, Zaria, Kaduna State, were invited to participate in the study; adults who were too sick due to an illness or mental issue throughout the research period, such as infectious disorders or Alzheimer's, were not considered eligible. The sample size was calculated to be 385 and a systematic random sampling technique was used. Ethical approval was obtained from ethical committees of Universiti Putra Malaysia (JKEUPM-2019-036) and ABUTH, Zaria, Kaduna State, Nigeria (ABUTHZ/G30/2019). A questionnaire in English was used to collect the information. A variety of demographic, belief, and ART compliance questionnaires were filled out by those who took part.

The Beliefs about Medicines Questionnaire (BMQ) is a questionnaire that was adapted from Horne et al. [15]. The BMQ is made up of two five-item scales that examine patients' beliefs about the need for prescribed medication to control their disease (five statements) as well as their concerns about possible side effects (five statements). Answers on a 5-point Likert scale are provided for each statement (strongly disagree, disagree, uncertain, agree and strongly agree). The responses are ranked from 1 (strongly disagree) to 5 (strongly agree). Five items make up the specific-necessity and specific-concerns measures, with values ranging from 5 to 25. The points on each scale are added together to produce a scale score. Higher scores imply a stronger belief in the scale's concepts. Higher specific-necessity scores indicate a greater sense of personal need for the medicine now and in the future. Higher specific-concerns scores indicate more concern about the medication's potential side effects.

The Centre for Adherence Support Evaluation (CASE) of the New York Academy of Medicine (NYAM) developed the CASE adherence tool. CASE adherence tool is three adherence questions in all. Better adherence is indicated by a higher composite score. The results of the CASE adherence instrument were combined to produce a combined measure that varied from 3 to 16 points. Non-adherent patients had an index score of less than 10, while those with a score of ≥ 10 were considered adherent [26].

Statistical analysis

Statistical analysis was conducted using SPSS version 25 (IBM Corp., Armonk, NY, USA). For categorical data, frequency and percentage were employed, whereas continuous data was represented by mean and standard deviation (SD). Pearson's chi-square or Fisher exact tests were used to explore the connection between adherence to ART as a dependent variable and the independent variables. To identify food insecurity and other characteristics related with adherence to ART, a multivariate regression analysis was undertaken, starting with basic logistic regression with (p = 0.25) [27]. Using an entry variable selection, multiple logistic regression with a 95% confidence interval was performed. The alpha 0.05 significance level was used.

## Results

There were 385 participants, 260 (67.5%) of whom were female and 125 (32.5%) male. The majority of the respondents (196, 50.9%) were between the ages of 49 and 64, and more than half of them (54.8%) were of Hausa ethnicity. Muslims made up the majority of the responders (70.6%). Over half of the respondents (57.7%) have a monthly income of more than 40,000 naira (USD 97.23). About 23.1% of those polled were jobless or retired, 49.1% were self-employed or business owners, and 27.8% worked for the government. Over half of the respondents (60.3%) were married, while 19% were single and 20.8% were divorced or widowed (Table 1).

**Table 1:** Distribution of respondents by socio-demographic characteristics

Variables	n	%	Mean ± SD
Age (Years)			40.77 ± 9.94
18–33	72	18.7	
34–48	117	30.4	
49–64	196	50.9	
Gender			
Male	125	32.5	
Female	260	67.5	
Ethnicity			
Hausa	211	54.8	
Yoruba	65	16.9	
Igbo	53	13.8	
Others	56	14.5	
Religion			
Christianity	113	29.4	
Islam	272	70.6	
Educational level			
No formal	32	8.3	
Primary school	66	17.1	
Secondary school	114	29.6	
Tertiary	173	44.9	
Occupation			
Unemployed/retired	89	23.1	
Self-employed/business	189	49.1	
Government employed	107	27.8	
Marital status			
Single	73	19.0	
Married	232	60.3	
Widowed or divorced	80	20.8	

Table 2 shows that about 19.7% of the respondents strongly disagree that their health at present depends on ART medicines and 23.4% of the respondents disagreed. About 39.0% of the respondents agreed that their lives would be impossible without ART, while 24.9% of the respondents disagreed. About 44.4% of the respondents agreed that without ART medication they would be very ill and 22.3% of the respondents disagreed. More than half (53.2%) of the respondents agreed that health in the future will depend on ART and 20% of the respondents disagreed. More than half (62.1%) of the respondents agreed that ART medication protects them from becoming worse while 15.3% disagreed. However, 25.7% of the respondents strongly agreed that ART medication worries them and 12.7% of the respondents strongly disagreed. More than half (59.2%) of the respondents agreed that they worry about the long-term effects of ART. About 25.7% of the respondents strongly agreed that ART is a mystery to them and 12.7% of the respondents strongly disagreed. About 25.7% of respondents strongly agreed that ART medication disrupts their lives while 28.1% of the respondents agreed. More than half (53.8%) of the respondents agreed that sometimes they worry about becoming too dependent on ART medication and about 19.7% of the respondents disagreed (Table 2). Higher specific-necessity scores indicate a stronger belief in the medication's personal requirement to sustain health now and in the future. More than half (55.8%) of the respondents had negative perceptions (beliefs) of personal need for ART medication and about 44.2% of the respondents had positive perceptions (beliefs) of personal need for ART medication.

**Table 2:** Beliefs and patient preference for medicine towards antiretroviral treatment (ART) (n=385)

	Strongly agree n (%)	Agree n (%)	Uncertain n (%)	Disagree n (%)	Strongly disagree n (%)
Specific-necessity					
My health at present depends on my ART medicines	99 (25.7)	108 (28.1)	12 (3.1)	90 (23.4)	76(19.7)
My life would be impossible without my ART medication	74 (19.2)	150 (39.0)	29 (7.5)	96 (24.9)	36(9.4)
Without my ART medication I would be very ill	74 (19.2)	171 (44.4)	17 (4.4)	86 (22.3)	37(9.6)
My health in the future will depend on my ART medication	56 (14.5)	205 (53.2)	11 (2.9)	77 (20.0)	37(9.4)
I'm prevented from getting worse by my ART treatment.	54 (14.0)	239 (62.1)	3 (0.8)	59 (15.3)	30 (7.8)
Specific-concerns					
Having to take ART medication worries me	99 (25.7)	99 (25.7)	42 (10.9)	96 (24.9)	49 (12.7)
I sometimes worry about the long-term effects of my ART medication	79 (20.5)	228 (59.2)	9 (2.3)	51 (13.2)	49(4.7)
My ART medication is mystery to me	99 (25.7)	99 (25.7)	42 (10.9)	96 (24.9)	49(12.7)
My ART medication disrupts my life	99 (25.7)	108 (28.1)	12 (3.1)	90 (23.4)	76(19.7)
I sometimes worry about becoming too dependent on my ART medication	63 (16.4)	207 (53.8)	6 (1.6)	76 (19.7)	33 (8.6)

Table 3 shows that the higher the specific-concerns score, the more concern there is about the medication's possible side effects. More than half of the respondents (57.7%) were less concerned about the ART medication's possible side effects and about 42.3% of the respondents had more concerns about the potential negative effects of the ART medication. Table 4 shows that about 46% of the patients were non-adherent. 

**Table 3:** Distribution of respondents’ belief towards antiretroviral treatment (ART) (n=385)

Variables	Scores	n	%	Mean ± SD
Specific-necessity scores				13±2.95
Negative perceptions of personal need for the medication	5-13	215	55.8	
Positive perceptions of personal need for the medication	14-22	170	44.2	
Specific-concerns scores				13±3.35
Fewer concerns about the potential negative effects of the medication	6-13	222	57.7	
More concerns about the potential negative effects of the medication	14-24	163	42.3	

**Table 4:** Prevalence of adherence to antiretroviral treatment (ART) of the respondents

Variables	Score	n	%
Good adherence	>10	208	54.0
Poor adherence	<10	177	46.0

Table 5 outlines the important characteristics that influence adherence to ART. Adherence to ART was found to be significantly related to age (ꭓ² 0 = 9.179, p < 0.05). A greater proportion of ART non-adherents was found in respondents who were 49-64 years old (59.7%) compared to those who were 18-33 (38.9%) and 34-48 (53.8%) years old. Besides, non-adherence to ART was significantly associated with educational level (ꭓ² = 8.458, p < 0.05). A higher proportion of ART non-adherence was found among respondents who had attained tertiary education (50.0%), compared to respondents who had attained no formal education (7.7%), primary (13.5%), and secondary education (28.8%). Occupation was associated with being adherent to ART (ꭓ² = 9.061, p < 0.05). A greater proportion of non-adherents to ART were found among the government-employed (60.7%) than the self-employed/business (56.6%) and unemployed (40.4%). Marital status was also significantly associated with adherents to ART (ꭓ² = 7.293, p < 0.05). Respondents who were divorced or widowed had a higher proportion of non-adherents to ART (66.3%) than those who were single (45.2%) and married (52.6%).

**Table 5:** Association between socio-demographic and adherence to antiretroviral treatment (ART)

Variables	Adhere n(%)	Non- adhere n (%)	ꭓ²	p-value
Age (Years)			9.179	0.010
18–33	44 (61.1)	28 (38.9)		
34–48	54 (46.2)	63 (53.8)		
49–64	79 (40.3)	117 (59.7)		
Gender			2.706	0.100
Male	65 (36.7)	60 (28.8)		
Female	112 (63.3)	148 (71.2)		
Ethnicity			2.865	0.413
Hausa	96 (54.2)	115 (55.3)		
Yoruba	25 (14.1)	40 (19.2)		
Igbo	28 (15.8)	25 (12.0)		
Others	28 (15.8)	28 (13.5)		
Religion			2.506	0.113
Christianity	59 (33.3)	54 (26.0)		
Islam	118 (66.7)	154 (74.0)		
Educational level			8.458	0.031
No formal	16 (9.0)	16 (7.7)		
Primary school	38 (21.5)	28 (13.5)		
Secondary school	54 (30.5)	60 (28.8)		
Tertiary	69 (39.0)	104 (50.0)		
Occupation			9.061	0.011
Unemployed/retired	53 (59.6)	36 (40.4)		
Self-employed/business	82 (43.4)	107 (56.6)		
Government employed	42 (39.3)	65 (60.7)		
Marital status			7.293	0.026
Single	40 (54.8)	33 (45.2)		
Married	110 (47.4)	122 (52.6)		
Widows and divorced	27 (33.8)	122 (52.6)		

Furthermore, respondents' beliefs about medicines were related with adherence to ART in a significant way. Both specific-necessity and specific-concerns for medication were significantly associated with adherence to ART (ꭓ² = 12.812, p < 0.05, ꭓ² = 12.826, p < 0.05). A higher proportion of non-adherence to ART was found in respondents who had negative perceptions (beliefs) of personal need for the medication (53.5%) than respondents who had positive perceptions (45.3%) of personal need for the medication to maintain health now and in the future. On the other hand, respondents who had more concerns about the potential negative effect of the medication had a higher proportion of non-adherence to ART (53.4%) compared to respondents who had fewer concerns (45.5%) about potential negative effects of the medication (Table 6).

**Table 6:** Association between belief in medicine and adherence to antiretroviral treatment (ART)

Variables	Adhere n (%)	Non-adhere n (%)	ꭓ²	p-value
Specific-necessity scores			12.812	0.050
Negative perceptions of personal need for the medication	100 (46.5)	115 (53.5)		
Positive perceptions of personal need for the medication	77 (54.7)	93 (45.3)		
Specific-concerns scores			12.826	0.048
Less concerns about the negative effects of the medication	101 (54.5)	121 (45.5)		
More concerns about the negative effects of the medication	76 (46.6)	87 (53.4)		

Table 7 shows that respondents who were self-employed were more likely to be ART non-adherents than those who were employed. When compared to unemployed participants, self-employed participants had a two-fold increase in chances (adjusted odds ratio (AOR) = 2.646, 95% CI: 1.335, 5.241). In addition, respondents who worked for the government had a 2.8-fold higher chance of not adhering to ART than those who did not (AOR = 2.842, 95% CI: 1.542, 5.240). Divorced or widowed respondents were also twice as likely as single or married respondents to stop using ART (AOR = 2.016, 95% CI: 1.111, 3.660). And respondents who had negative perceptions (beliefs) of personal need for ART medication to maintain health now and in the future were 1.5 times more likely to be non-adherent to ART than respondents who had positive perceptions (beliefs) of personal need for the ART medication to maintain health now and in the future (AOR = 1.525, 95% CI: 1.958-2.427). Also, respondents who had more concerns about the potential negative effects of the ART medication were 1.3 times more likely to be non-adherent to ART than respondents who had fewer concerns about the potential negative effects of the ART medication (AOR = 1.362, 95% CI: 1.751, 2.005). 

**Table 7:** Factors associated with adherence to antiretroviral treatment (ART)

Factors	B	SE	Adjusted Odds Ratio (AOR)	95CI	p-value
Occupation Unemployed	ref				
Self-employed/business	0.973	0.349	2.646	1.335–5.241	0.005
Government employed	1.045	0.312	2.842	1.542–5.240	0.001
Marital status Single	ref				
Married	0.204	0.368	1.226	0.596–2.520	0.580
Others	0.701	0.304	2.016	1.111–3.660	0.021
Specific-necessity					
Negative perceptions	0.422	0.237	1.525	1.958-2.427	0.045
Positive perceptions	ref				
Specific-concerns					
Fewer concerns	ref				
More concerns	0.871	0.255	1.362	1.751-2.005	0.048

## Discussion

The prevalence of ART adherence differs by country and location. Adherence to ART was 71.0% in Northern Tanzania and 62.2% in Ghana [28,29]. Antiretroviral therapy compliance was 88.2% in Ethiopia [30], while in Togo, 78.4% were adherent to ART and in South Africa, 69.0% were adherent to ART [31,32]. Furthermore, Eyassu et al. found that 77% of patients in other African nations, such as South Africa, were adhering to their treatment [33]. Roux et al. found that 61.0% of patients in the Centre area of Cameroon were adhering to ART, while Gatongi et al. found 68% baseline adherence in Ethiopia and 73.4% in Nigeria [34-36]. ART effectiveness is reduced and medication resistance is increased when patients do not adhere to their treatment [37]. In Nigeria, very few recent studies on ART adherence have been done and reported across the country. In examining the reported findings from different studies, compared to the findings of the present study, most of the findings reported higher suboptimal adherence levels, for instance the study in the South-South region in Niger Delta revealed 73.4% adherence, the study in Lagos (Southwest) revealed an adherence of 78.4% [36,38]. The study in Ile Ife reported 63% adherence, in the South Eastern region of Nigeria, 86.1% was reported as the adherence rate [39,40]. In the North Central, the study in Ilorin reported 70.8% adherence [41]. When compared to other regions in Nigeria, ART adherence was found to be lower among HIV respondents on ART in ABUTH, Zaria. The majority of patients interviewed in this study were non-adherent in aspects of forgetfulness and time, which could be attributed to a bad health sector and facilities, such as poor relationships with healthcare practitioners, long waiting times, and vast distance. These factors contributed to low adherence to ART in the current study compared to other studies done in other regions of Nigeria.

The findings of this study revealed that most of the respondents had attained a tertiary level of education. This is consistent with findings of Amoran and Martins et al. [42,43], in South West and Northern Nigeria where most of the respondents had a tertiary education. The current study revealed that half of the respondents who had low adherence to ART were those who attained tertiary education levels. This is consistent with findings by Hansana et al. and Pennap et al. [44,45], where most of the respondents who had low adherence to ART were respondents who attained tertiary education levels. When the relationship between educational attainment and adherence to ART was investigated, however, it was discovered to be substantial (ꭓ² = 8.458, p < 0.05). The current study was consistent with the previous studies conducted by Okunola et al. and Suleiman and Momo [23,36], which reveals there was a link between education and adherence to ART, according to the studies. In this study, most of the respondents who attained a tertiary level of education were non-adherent to ART, which may be related to the fact that these groups of HIV-positive people had demanding jobs that made it difficult for them to take the medication.

In the current study, one of the characteristics affecting adherence to ART was the respondents' profession. Government employees had a larger percentage of non-adherent respondents than self-employed/business owners or unemployed respondents. Suryana et al. found a significant relationship between ART compliance and occupational status [46]. Prah et al. found that occupation status was significantly linked with adherence to treatment [47]. According to Safira et al., compliance with ART was strongly linked to employment position [48]. The findings of the current study, on the other hand, were consistent with those of Okoronkwo et al., who found that the unemployed seem to be more likely than the employed or self-employed to adhere to ART [49]. Saha et al. also explored compliance to highly active ART and discovered that employed respondents were more non-adherent to ART than unemployment respondents [50]. Busy work schedules and/or forgetfulness were the most likely reasons for non-adherence among HIV-positive employed or self-employed adults.

In this study, married people made up the majority of the respondents. Divorced or widowed respondents were assigned to the "other" category. Divorced and widowed respondents were two times more likely than married respondents to stop using ART. In comparison to other marital situations, adherence was lowest among divorced people with a lower number of women adhering. The fact that most married women had already told their sexual partners about their HIV status was a crucial factor in their high adherence [32]. Single, married, cohabiting, and divorced/separated were the four groups used in Bam et al. study [51]. The current study, on the other hand, improves on this by looking into the chance of becoming a widow. Bam et al. looked at the end result of marital status on adherence to ART and when compared to divorced, separated, single, and other marital statuses, they discovered that 39 married people accounted for the biggest proportion of participants who adhered to ART (83.3%) in a descriptive cross-sectional study to analyses barriers and facilitators of ART adherence [51]. It implied that HIV-positive people's adherence was influenced by their spouses.

Non-adherence to ART was 1.5 times more in respondents who had a weak view of personal need for ART medicine to preserve health now and in the future than in respondents who had a strong perception of personal need for ART medication to maintain health now and in the future. Also, those who were very concerned about the potential bad effects of the ART medicine were 1.3 times more likely to be non-adherence to ART than those who were less concerned. These results were consistent with other studies that revealed that beliefs in medicine are associated with compliance to ART [52-54]. Medicine beliefs, whether positive or negative, have an impact on medication usage. In the current study's interviews, some of the participants thought they were going to die of HIV because they don't believe the medication can assist or help improve their health. For some, this idea stemmed from personal experiences, such as witnessing a family member die from an AIDS-related illness. Because HIV is incurable, some respondents believed that taking treatment would be futile because they would die from it. Taking HIV medication on a daily basis did not mesh with these people's perceptions of HIV as a terminal disease. Many of the people who responded to the survey were devastated and overwhelmed by their HIV diagnosis, and many found it difficult to agree with the fact that they were HIV positive while still committing to lifelong treatment. Respondents said they had trouble processing the information they were given in this context. They did not have enough time to express their concerns about ART to their doctor, so they began treatment with great reservations about taking it, resulting in non-adherence to the medicine.

There are several limitations in this study, Firstly, the cross-sectional nature of the data limited the study's capacity to make any causal inferences regarding the data. Secondly, self-reporting of respondents was used, which was susceptible to recalling prejudice and overestimating adherence. Thirdly, there were limited current studies on belief in medicine associated with adherence to ART in Nigeria as compared to other African countries. Therefore, the recent study failed to see if there are improvements in adherence to ART over the years and patient belief. More research studies on adherence and patient belief should be conducted from time to time at Federal Medical Center to assess the level of adherence of HAART patients and also plan interventions on how to improve adherence levels among the patients. According to the findings of this study, there was a low prevalence of adherence to ART. As a result, the strategies used to encourage compliance must be expanded. More education and counselling are likely to be required such as educational programs and following up the patients even after leaving the clinic. Lastly, during treatment at the ART clinic, clinicians should provide counselling on the importance of adherence to their medication. Therefore, to overcome these barriers, interventions at the village or district level, resources for individuals and communities, such as community care centers and home-based care, must be expanded and also implement programs on television to increase awareness and educate people on their beliefs regarding the medication. 

## Conclusions

In summary, occupation, marital status and belief in medicine were associated with adherence to ART among HIV adults. Workplace regulations must be in place to assist persons with chronic conditions such as HIV/AIDS in sticking to their treatment regimens. Participants who had less belief and more concerns towards ART medication were more noncompliant with ART; this could be a result of poor understanding of the importance of ART medication and cultural beliefs. Therefore, during adherence counseling, healthcare personnel should address respondents' false views and fears regarding ART medication in order to strengthen proper information and the benefits of ART.
